# A CT-based radiomics model for predicting renal capsule invasion in renal cell carcinoma

**DOI:** 10.1186/s12880-022-00741-5

**Published:** 2022-01-30

**Authors:** Lu Yang, Long Gao, Dooman Arefan, Yuchuan Tan, Hanli Dan, Jiuquan Zhang

**Affiliations:** 1 Department of Radiology, Chongqing University Cancer Hospital and Chongqing Cancer Institute and Chongqing Cancer Hospital, Chongqing, 400030 People’s Republic of China; 2 Department of Radiology, University of Pittsburgh, Pittsburgh, PA 15213 USA; 3 College of Computer, National University of Defense Technology, Changsha, 410073 China

**Keywords:** Renal cell carcinoma, Capsule invasion, Computed tomography, Radiomics; machine learning

## Abstract

**Background:**

Renal cell carcinoma (RCC) is a heterogeneous group of kidney cancers. Renal capsule invasion is an essential factor for RCC staging. To develop radiomics models from CT images for the preoperative prediction of capsule invasion in RCC patients.

**Methods:**

This retrospective study included patients with RCC admitted to the Chongqing University Cancer Hospital (01/2011–05/2019). We built a radiomics model to distinguish patients grouped as capsule invasion versus non-capsule invasion, using preoperative CT scans. We evaluated effects of three imaging phases, i.e., unenhanced phases (UP), corticomedullary phases (CMP), and nephrographic phases (NP). Five different machine learning classifiers were compared. The effects of tumor and tumor margins are also compared. Five-fold cross-validation and the area under the receiver operating characteristic curve (AUC) are used to evaluate model performance.

**Results:**

This study included 126 RCC patients, including 46 (36.5%) with capsule invasion. CMP exhibited the highest AUC (AUC = 0.81) compared to UP and NP, when using the forward neural network (FNN) classifier. The AUCs using features extracted from the tumor region were generally higher than those of the marginal regions in the CMP (0.81 vs. 0.73) and NP phase (AUC = 0.77 vs. 0.76). For UP, the best result was obtained from the marginal region (AUC = 0.80). The robustness analysis on the UP, CMP, and NP achieved the AUC of 0.76, 0.79, and 0.77, respectively.

**Conclusions:**

Radiomics features in renal CT imaging are associated with the renal capsule invasion in RCC patients. Further evaluation of the models is warranted.

## Background

Renal cell carcinoma (RCC) is a heterogeneous group of kidney cancers comprising many histologic subtypes, with clear cell histology being the most common subtype [1, 2]. RCC is the seventh most common cancer leading to death of 140,000 patients worldwide every year [3–5]. According to the tumor-node-metastasis (TNM) staging criteria for RCC in the eighth edition American Joint Committee on Cancer (AJCC) guideline [6], renal capsule invasion is an essential factor distinguishing the T2 and T3 stages.

The renal capsule is a firm fibrous layer surrounding the kidney and is covered by a thick layer of perinephric adipose tissue. The fibrous layer can protect tumor from seeding or spreading to the adjacent tissue, thus a capsular invasion is an early sign of cancer spreading and reflects a tumor’s aggressiveness. Several studies [7–9] showed that the prognosis of RCC patients with renal capsule invasion was poorer than those without the invasion. A recent study showed that the existence and extent of renal capsule invasion are associated with prognosis, while lymphovascular invasion was not [10]. Another study on surgically treated stages I and II RCC patients showed that patients with invasion but no penetration had an adverse prognostic outcome [11]. Therefore, the prediction of capsular invasion may be an essential factor for the staging of RCC and is vital to the selection of appropriate treatments for RCC patients.

The visual assessment of capsule invasion at imaging, however, can be challenging because RCC patients with capsule invasions may or may not invade the renal perirenal fat space [12–15]. Recent work proposed an approach for scoring renal capsule invasion on surgical specimens [16]. Still, non-invasive methods such as computed tomography (CT) imaging for assessing potential capsule invasion may provide a pre-surgical prediction of capsule invasion for RCC patients and guide the surgical planning.

Radiomics is a computational method to extract massive quantitative features from medical images for investigating clinical outcomes [17, 18]. Previous applications of radiomics in RCC mainly focused on three aspects: (1) identifying malignant renal tumors from benign lesions [19, 20]; (2) predicting the Fuhrman grade using different imaging-based models [21, 22]; and (3) differentiating different subtypes of renal cancer [19, 23]. To the best of our knowledge, few reported studies have examined the prediction of capsule invasion in RCC patients using radiomics. The purpose of this study was to develop radiomics models from CT images for the preoperative prediction of capsule invasion in RCC patients, aiming to improve the clinical management of patients with RCC.

## Methods

### Study cohort

This study was a retrospective study and was approved by the ethics committee of the Chongqing University Cancer Hospital (CZLS2021068-A). The requirement for informed consent was waived by the ethics committee of the Chongqing University Cancer Hospital. The study included patients admitted to the Chongqing University Cancer Hospital and diagnosed with RCC from January 2011 to May 2019. The inclusion criteria were (1) pathologically confirmed RCC after partial or radical nephrectomy surgery and (2) complete CT scan acquired within 2 weeks before surgery.

A histopathological evaluation was performed with hematoxylin and eosin staining for all patients, along with immunohistochemistry when needed. The tumor histological findings were classified according to the WHO 2004 system [24]. An associate chief pathologist with 10 years of experience and specialized in renal pathology re-examined all specimens. According to the pathological results, the specimens were categorized into two groups: capsule invasion and non-capsule invasion.

### Data collection

All data of the patients included in this study were collected from the Integrated Electronic Medical Record System of the Chongqing University Cancer Hospital, including age, sex, tumor location, maximal diameter of the tumor, Furhman stage, and lymph node metastasis.

### CT imaging parameters

All patients underwent multi-phase enhanced CT scanning. Three different CT systems (Philips Brilliance CT 64, SOMATOM Definition AS, and SOMATOM Drive) with comparable clinical operations were used on patients upon availability. The majority of patients (n = 107; 85%) were scanned using the Philips Brilliance CT 64. Table 1 presents the specific imaging parameters.

**Table 1:** CT scanning parameters and information

Imaging parameters	Philips brilliance CT	SOMATOM definition AS	SOMATOM drive
Detector collimation, mm	64 × 0.625	128 × 0.6	128 × 0.6
Pitch	1.016	0.6	0.6
Tube voltage, kV	120	120	120
Tube current	250mAs	CARE Dose4D	CARE Dose4D
FOV, cm	35 40	35 40	35 40
Reconstruction section thickness, mm	2	2	2
Slice spacing, mm	2	2	2
Year of installation	2008	2018	2019
Patients numbers, n	107	13	6

*FOV* field of view, *CT* computed tomography

For contrast-enhanced imaging, iodixanol alcohol 80–90 mL (320 mg/mL) was injected into the elbow vein with a high-pressure syringe at a rate of 2.5–4.0 mL through a power injector. The scanning started from the top of the diaphragm to the level of the iliac wing. The CT images were acquired at three different scanning phases: (1) phase 1, unenhanced phase (UP), before the injection of the contrast agent; (2) phase 2, corticomedullary phase (CMP), 7 s after aortic enhancement exceeded 150 HU compared to baseline; and (3) phase 3, nephrographic phase (NP), 20 s after CMP.

### Tumor segmentation

The tumors in the UP, CMP, and NP images were manually segmented by a certified associate chief radiologist with 10 years of experience, using the Philips Radiomics Tool(Philips Healthcare, Shanghai, China). The largest cross-sectional region of the tumor was selected for segmentation. Then, another senior radiologist (10 years of experience) reviewed the selection to confirm or make corrections to the initial segmentation results. To evaluate the effects of the tumor margin regions, an extended area of 3 mm adjacent to the tumor boundary was automatically generated by image processing algorithms to extract the radiomics features of the peritumor region. The 3-mm margin was determined based on the clinical experience and considerations of the two expert radiologists.

### Feature extraction, selection, and classification

**Fig. 1:**
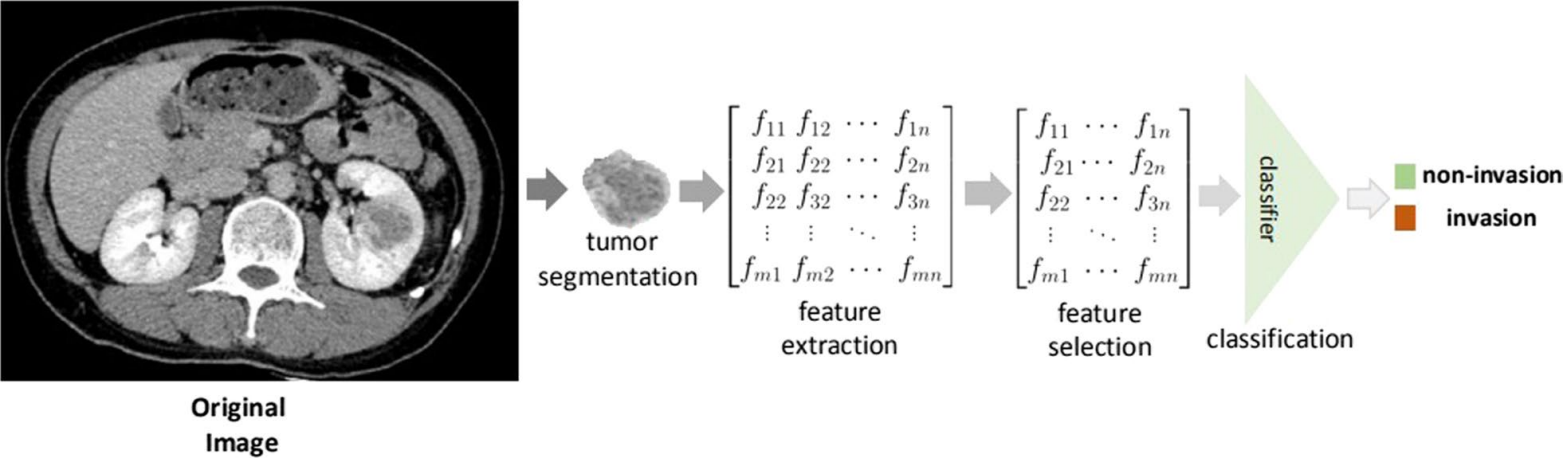
The pipeline of the proposed radiomics modeling. First, tumor was manually segmented in CT images. Second, the features were extracted using Pyradiomics software. Third, the features were selected using the least absolute shrinkage and selection operator (LASSO) method. Finally, binary-class classification was performed with different classifiers

As seen in Fig. 1, a standard radiomics pipeline was followed. Pyradiomics [25] was used to extract 119 radiomics features, including morphological and texture features. These features were extracted from UP, CMP, and NP separately. The effects of every single scanning phase were first evaluated. Then, considering that the imaging features extracted from different scanning phases may be complementary, we also assessed the combination of these features extracted from different scanning phases: UP + CMP and UP + CMP + NP.

To evaluate the effects of the tumor regions and the tumor’s margin regions, radiomics models were built using features from the tumor region alone, the marginal region alone, and their combination. Two “combination” modes were evaluated: (1) combining radiomics features separately extracted from tumor and marginal area (marked with “T + M”); and (2) extracting features directly from the combined/integrated region of the tumor and margin (denoted by “whole region”).

### Statistical analysis

The least absolute shrinkage and selection operator (LASSO) algorithm [26] was used to select a subset of the most related features for machine learning modeling. The effects of five machine learning classifiers were tested: support vector machine (SVM) with radial basis function (RBF) kernel, linear discriminant analysis (LDA), k-nearest neighbor (kNN) (k = 5), logistic regression (LR), and forward neural network (FNN), for the binary classification of “capsule invasion” versus “capsule non-invasion”. The FNN includes three layers: input, hidden, and output. The MATLAB R2018a Statistics (MathWorks, Natick, MA, USA) with its Machine Learning Toolbox was used to implement the classifiers. Considering the size of the cohort, patient-wise fivefold cross-validation was performed to evaluate the classification effects. Receiver operation characteristic (ROC) curve analyses and the area under the ROC curve (AUC) were used as the model performance metric. The continuous data were presented as median (range) and were analyzed using the Mann–Whitney U-test. Categorical data were presented as numbers (percentages) and were analyzed using the chi-square test. Two-sided *p* < 0.05 was considered statistically significant.

## Results

### Patient characteristics

This study included 126 patients with RCC; 46 had capsule invasion, and 80 had no capsule invasion. Table 2 summarizes the characteristics of the patients. The median age was 57.5 years. There were 70 males and 56 females, and the proportion of males was lower in the capsule invasion group (43.5% vs. 62.5%, *p* = 0.039). The number of tumors located on the left and right sides was similar. The tumor size ranged from 1.2 to 16 cm, and the tumors were smaller in the capsule invasion group (5.6 vs. 6.0 cm, *p* = 0.046). The patients in the capsule invasion group had more advanced stages (*p* = 0.002). According to pathology, only nine (7.1%) patients out of 126 had lymph node metastasis.

**Table 2:** Characteristics of the patients

Characteristics	Total (n = 126)	With capsule invasion (n = 46)	Without capsule invasion (n = 80)	*p* Value
Age, years, median (range)	57 (28, 87)	59 (43, 85)	56 (28, 87)	0.073
Sex, n (%)				
Male	70 (55.6)	20 (43.5)	50 (62.5)	0.039
Female	56 (44.4)	26 (56.5)	30 (27.5)	
Tumor location, n (%)				
Left	63 (50)	26 (56.5)	37 (46.2)	0.383
Right	63 (50)	20 (43.5)	43 (53.8)	
Max diameter (cm)	5.8 (1.2, 16)	5.6 (1.5, 12)	6.0 (1.2, 16)	0.046
Furhman stage, n (%)				
I	19 (15.1)	0	19 (23.8)	0.002
II	72 (57.1)	30 (65.2)	42 (52.5)	
III	23 (18.3)	7 (15.2)	16 (20.0)	
IV	12 (9.5)	9 (19.6)	3 (3.8)	
Lymph node metastasis, n (%)	9 (7.1)	4 (8.7)	5 (6.3)	0.585

### Classification results

**Fig. 2:**
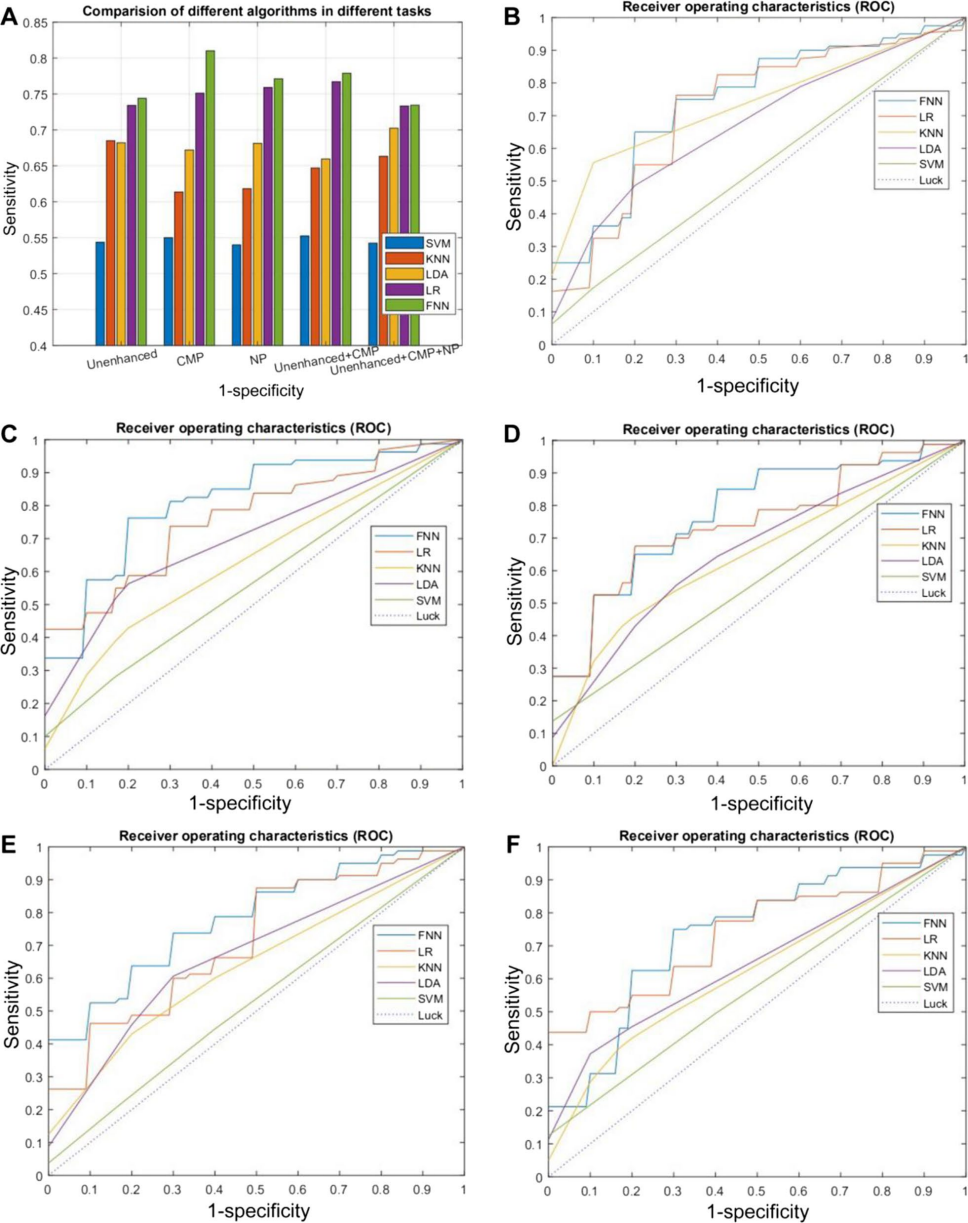
The receiver operating characteristic (ROC) curves and area under the curve (AUC) of five different machine learning algorithms for the classification of capsule invasion versus non-invasion in different CT imaging phases. **A** The comparison of AUCs of different machine learning algorithms. **B**–**F** The ROC curves in different imaging phases (**B**: the unenhanced phase; **C**: corticomedullary phase (CMP); **D**: nephrographic phase (NP); **E**: unenhanced + CMP; **F**: unenhanced + CMP + NP). *FNN* forward neural network, *LR* logistic regression, *KNN* k-nearest neighbor, *LDA* linear discriminant analysis, *SVM* support vector machine

Figure 2 shows the classification results of the five classifiers on different sets of features extracted from the images and the corresponding ROC curves. Overall, FNN performed the best among the classifiers. For the three different scanning phases of the tumor region, CMP exhibited the highest AUC (AUC = 0.81) compared to UP and NP. In general, the performance dropped in FNN when combining features from different scanning phases.

Based on the results mentioned above, FNN was used as the primary model for the subsequent analyses with features extracted from different regions. Table 3 listed the features selected for the UP, CMP, and NP in the tumor region As shown in Table 4, the tumor region achieved slightly higher AUCs than the marginal area in CMP and NP. And the AUC of the features extracted from the tumor or marginal regions separately is generally higher than from the combined regions. For the UP, the best result was obtained from the marginal area (AUC = 0.80). And the combination of the features extracted from the UP and CMP generally achieved lower AUCs than using either UP or CMP alone. In addition, the AUC decreases significantly when we combined the features extracted from all three scanning phases compared to using the features extracted from UP, CMP and NP alone.

**Table 3:** The features selected across all five folds

ROI	Selected features
UP	Original_firstorder_10Percentile
	Original_firstorder_Kurtosis
	Original_firstorder_Median
	Original_glcm_ClusterProminence
	Original_glszm_LargeAreaLowGrayLevelEmphasis
	Original_glszm_SizeZoneNonUniformity
	Original-glszm_SizeZoneNonUniformityNormalized
	Original_ngtdm_Busyncss
	Original_ngtdm_Contrast
	Original_shape_Maximum2DDiameterColumn
	Original_shape_Maximum2DDiamcterRow
CMP	Original_glszm_LargeArcaHighGrayLevelEmphasis
	Original_ngtdm_Complexity
	Original_shape_Elongation
	Original_shape_Maximum2DDiameterRow
	Original_shape_SurfaceVolumeRatio
NP	Original_glcm_MCC
	Original_gldm_DependenceVariance
	Original_glszm_ZoncEntropy
	Original_ngtdm_Complexity
	Original_shape_Maximum2DDiameterRow
	Original_shape_SurfaceVolumeRatio

*ROI* region of interest, *UP* unenhanced phase, *CMP* corticomedullary phase, *NP* nephrographic phase

**Table 4:** AUCs of the FNN classifier on using different regions, CT imaging phases, and their combinations

Region	UP	CMP	NP	UP + CMP	UP + CMP + NP
Tumor	0.74	***0.81***	**0.77**	**0.78**	0.73
Marginal region	***0.80***	0.73	0.76	0.78	0.74
T + M	0.79	0.77	0.76	0.77	**0.75**
Whole region	0.78	0.75	0.76	0.72	0.72

*UP* unenhanced phase, *CMP* corticomedullary phase, *NP* nephrographic phase, *T* tumor, *M* marginal region, *AUC* area under the curve, *FNN* forward neural network

The number in bold is the best performance in each column. The number in italic is the best performance in each row

### Robustness evaluation of results

**Fig. 3:**
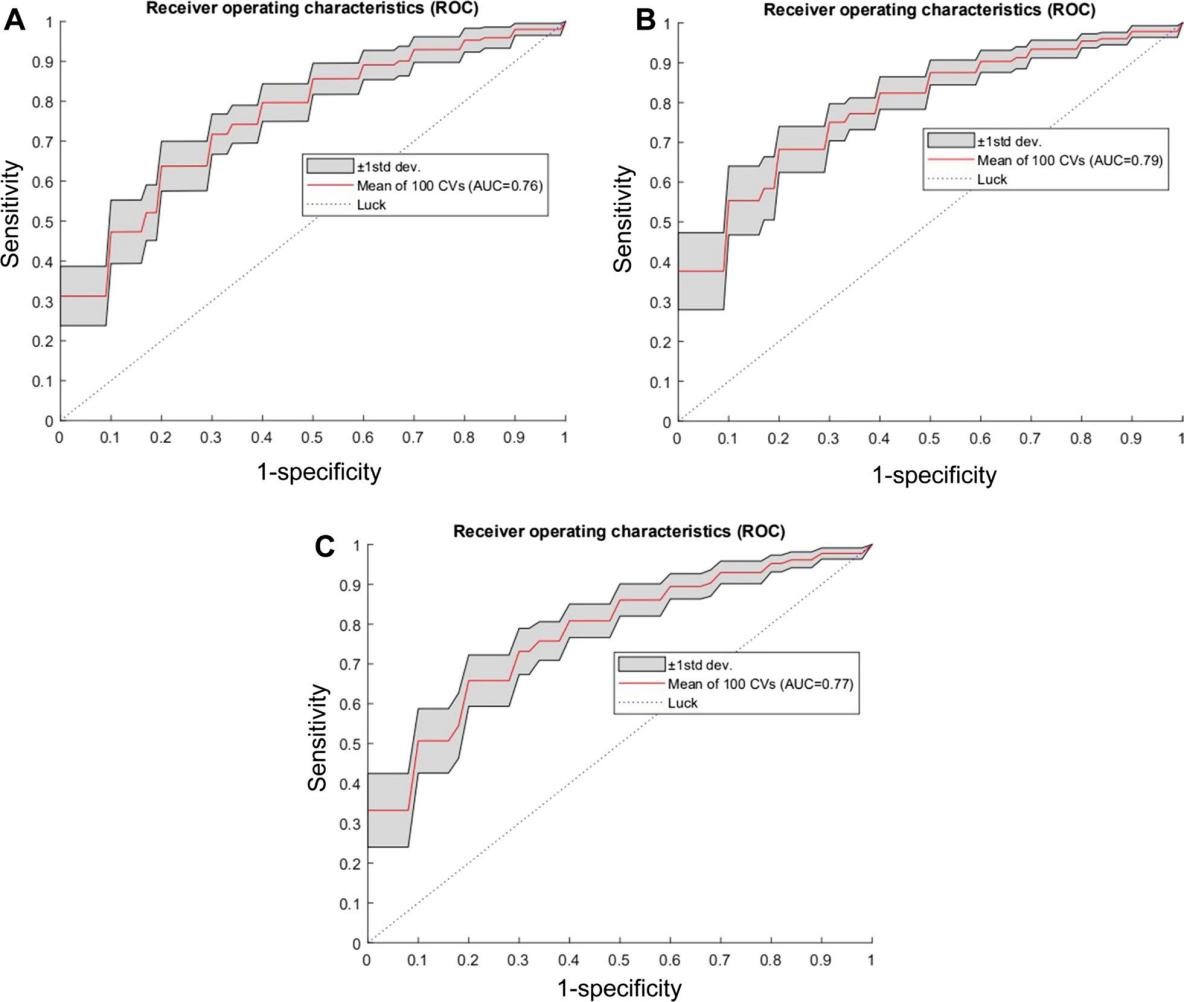
Robustness analysis of the forward neural network (FNN) for the classification of capsule invasion vs non-invasion. **A** Unenhanced phase. **B** Corticomedullary phase (CMP). **C** Nephrographic phase (NP). *CV* coefficient of variation, *AUC* area under the curve

To further evaluate the robustness of the FNN classifier, the algorithm was performed 100 times (each time all the cases were randomly split to form 5 folds for cross-validation) to determine the mean and standard deviation of the ROC curves for the UP, CMP, and NP (Fig. 3). Even when considering the lower standard deviation, the AUCs remained well above 0.500. The average ROC curve of the 100-round fivefold cross-validation on the UP, CMP, and NP obtained by the FNN classifier achieved AUCs of 0.76, 0.79, and 0.77, which were similar (*p* < 0.05) to the corresponding one-round result 0.74, 0.81, and 0.77, showing the stability of the FNN classifier and the radiomics model.

## Discussion

In this study, we developed a radiomics model from CT images for the preoperative prediction of capsule invasion in RCC patients. The results show an association between radiomics features in renal CT imaging and the renal capsule invasion of RCC, and, in particular, it shows that the characteristics of tumors at the CMP stage are predictive of the capsule's invasion.

The results of this study showed that the radiomics features separately extracted from the tumor or marginal regions are generally more predictive than the features extracted from the combined regions (i.e., “T + M” or the “whole region”). This indicates the LASSO method may be able to select better-performing feature combinations to achieve the higher classification performance. Previous studies have studied different features among tumor, peritumoral, and necrosis areas [27–30]. Therefore, in this study, the two type of regions (marginal vs. “whole region” and”T + M”) yield different features because of the different nature of the regions (malignant vs. non-malignant), and it is possible that, to certain extent, the features of the non-malignant area attenuate those of the malignant region. Nevertheless, the peritumoral region may carry useful information for diagnosis and characterization of various tumors [27–30], and future studies are needed to examine the effects of these different regions.

In addition, as observed for the whole versus individual T and M results, the combination of the features extracted from the UP and CMP phase showed overall worse performance than using either UP or CMP alone. It suggests that these two scanning phases may have some conflicting features for FNN to effectively learn patterns. Likewise, the model performance decreases significantly when we combine the features extracted from all three scanning phases compared to using the features extracted from UP, CMP and NP alone. This may further imply the increasing redundancy of information conveyed by these mixed features, making the feature selection procedure less effective. Of note, the UP phase achieved higher performance than CMP and NP as well as their combinations. This suggests that contrast-enhanced imaging may not be necessarily beneficial for the detection of capsule invasion. Yet, previous studies have shown various results regarding the effects of enhanced/unenhanced imaging [31–33]. This question remains to be further examined on larger datasets.

This study shows no significant association between RCC capsule invasion and age, tumor localization, and lymph node metastasis. There is moderate association between the Fuhrman stage and capsule invasion, as expected, based on the definition of the Fuhrman stages [7, 9, 21].

While previous work used simple qualitative imaging signs [9, 12, 15], this study applied quantitative radiomics methods to predict capsule invasion in RCC using unenhanced and contrast-enhanced imaging and their combinations. Using the FNN classifier and extracting features from the tumor region in the CMP achieved the highest AUC, reaching 0.81. The developed radiomics models can aid in staging and preoperative prognosis, and improve upon radiologists’ subjective interpretation of CT images. The radiomics model will be useful for patient prognostication before surgery, which has an important clinical value because the prognosis of RCC patients is currently assessed by the TNM staging system after surgical treatment, such as using radical or partial nephrectomy [15].

Our study has some limitations. First, the sample size was small, and this is a single-center study. Further evaluation of the models and findings are warranted on larger datasets. Second, the radiomics models use two-dimensional images instead of three-dimensional CT scan, so its performance needs further evaluation on three-dimensional data [17]. In addition, the tumor segmentation was performed manually that may introduced user-dependence and variations. Fully automated segmentation will be ideal in future work. Finally, a selection bias cannot be entirely avoided because this work is a retrospective study.

## Conclusions

In conclusion, preoperative CT-based radiomics features are shown to be associated with renal capsule invasion of RCC. Renal capsule invasion may be characterized by radiomics of unenhanced CT imaging and the CMP images. Compared to traditional visual assessment of images, the radiomics model may provide a tool to aid assessment of whether a capsule is invaded, and thus to better inform clinical prognosis and patient management. Further evaluation of our findings is warranted on large datasets in future work.

## Data Availability

The datasets used and/or analyzed during the current study are available from the corresponding author on reasonable request.

## Abbreviations

RCC: Renal cell carcinoma
UP: Unenhanced phases
CMP: Corticomedullary phases
NP: Nephrographic phases
AUC: Area under the receiver operating characteristic curve
FNN: Forward neural network
TNM: Tumor-node-metastasis
AJCC: American Joint Committee on Cancer
CT: Computed tomography
SVM: Support vector machine
RBF: Radial basis function
LDA: Linear discriminant analysis
kNN: K-nearest neighbor
LR: Logistic regression
ROC: Receiver operation characteristic

## References

[1] Moch H, Humphrey PA, Ulbright TM, Reuter V (2016). WHO classification of tumours of the urinary system and male genital organs.

[2] NCCN Clinical Practice Guidelines in Oncology (NCCN Guidelines). Kidney Cancer. Version 1.2021. Fort Washington: National Comprehensive Cancer Network; 2020.

[3] Capitanio U, Montorsi F (2016). Renal cancer. Lancet.

[4] Bray F, Ferlay J, Soerjomataram I, Siegel RL, Torre LA, Jemal A (2018). Global cancer statistics 2018: GLOBOCAN estimates of incidence and mortality worldwide for 36 cancers in 185 countries. CA Cancer J Clin.

[5] Siegel RL, Miller KD, Jemal A (2020). Cancer statistics, 2020. CA Cancer J Clin.

[6] Gallardo E, Mendez-Vidal MJ, Perez-Gracia JL, Sepulveda-Sanchez JM, Campayo M, Chirivella-Gonzalez I (2018). SEOM clinical guideline for treatment of kidney cancer (2017). Clin Transl Oncol.

[7] Song T, Yin Y, Liao B, Zheng S, Wei Q (2013). Capsular invasion in renal cell carcinoma: a meta-analysis. Urol Oncol.

[8] Qin X, Dingwei Y, Yao XD, Zhang S, Zhu Y, Zhang HL (2009). Role of renal capsular involvement status in renal cell carcinoma. China Oncol.

[9] Klatte T, Chung J, Leppert JT, Lam JS, Pantuck AJ, Figlin RA (2007). Prognostic relevance of capsular involvement and collecting system invasion in stage I and II renal cell carcinoma. BJU Int.

[10] Choosakul S, Harinwan K, Chirapongsathorn S, Opuchar K, Sanpajit T, Piyanirun W (2018). Comparison of normal saline versus Lactated Ringer's solution for fluid resuscitation in patients with mild acute pancreatitis. A randomized controlled trial. Pancreatology.

[11] May M, Brookman-Amissah S, Roigas J, Gilfrich CP, Pflanz S, Hoschke B (2010). Evaluation of renicapsular involvement in Stages I and II renal cell carcinoma from the morphological and prognostic point of view. Urol Oncol.

[12] Jeong IG, Jeong CW, Hong SK, Kwak C, Lee E, Lee SE (2006). Prognostic implication of capsular invasion without perinephric fat infiltration in localized renal cell carcinoma. Urology.

[13] Bonsib SM (2005). T2 clear cell renal cell carcinoma is a rare entity: a study of 120 clear cell renal cell carcinomas. J Urol..

[14] Zhang H, Wu Y, Xue W, Zuo P, Oesingmann N, Gan Q (2017). Arterial spin labelling MRI for detecting pseudocapsule defects and predicting renal capsule invasion in renal cell carcinoma. Clin Radiol.

[15] Zhang Y, Tian H, Zhang S, Zhang Q, Wu X (2018). Multislice spiral computed tomography signs of invasion of the renal capsule by renal cell carcinoma. Medicine (Baltimore).

[16] Snarskis C, Calaway AC, Wang L, Gondim D, Hughes I, Idrees MT (2017). Standardized reporting of microscopic renal tumor margins: introduction of the renal tumor capsule invasion scoring system. J Urol.

[17] van Timmeren JE, Cester D, Tanadini-Lang S, Alkadhi H, Baessler B (2020). Radiomics in medical imaging-"how-to" guide and critical reflection. Insights Imaging.

[18] Song J, Yin Y, Wang H, Chang Z, Liu Z, Cui L (2020). A review of original articles published in the emerging field of radiomics. Eur J Radiol.

[19] Coy H, Hsieh K, Wu W, Nagarajan MB, Young JR, Douek ML (2019). Deep learning and radiomics: the utility of Google TensorFlow Inception in classifying clear cell renal cell carcinoma and oncocytoma on multiphasic CT. Abdom Radiol (NY).

[20] Zhou L, Zhang Z, Chen YC, Zhao ZY, Yin XD, Jiang HB (2019). A Deep learning-based radiomics model for differentiating benign and malignant renal tumors. Transl Oncol.

[21] Ding J, Xing Z, Jiang Z, Chen J, Pan L, Qiu J (2018). CT-based radiomic model predicts high grade of clear cell renal cell carcinoma. Eur J Radiol.

[22] Shu J, Wen D, Xi Y, Xia Y, Cai Z, Xu W (2019). Clear cell renal cell carcinoma: Machine learning-based computed tomography radiomics analysis for the prediction of WHO/ISUP grade. Eur J Radiol.

[23] Yin Q, Hung SC, Rathmell WK, Shen L, Wang L, Lin W (2018). Integrative radiomics expression predicts molecular subtypes of primary clear cell renal cell carcinoma. Clin Radiol.

[24] Eble JN, Sauter G, Epstein JI, Sesterhenn IA (2004). WHO classification of tumours of the urinary system and male genital organs.

[25] van Griethuysen JJM, Fedorov A, Parmar C, Hosny A, Aucoin N, Narayan V (2017). Computational radiomics system to decode the radiographic phenotype. Cancer Res.

[26] Tibshirani R (2011). Regression shrinkage and selection via the lasso: a retrospective. J R Stat Soc B.

[27] Pei L, Vidyaratne L, Rahman MM, Iftekharuddin KM (2020). Context aware deep learning for brain tumor segmentation, subtype classification, and survival prediction using radiology images. Sci Rep.

[28] Sun Q, Lin X, Zhao Y, Li L, Yan K, Liang D (2020). Deep learning vs. radiomics for predicting axillary lymph node metastasis of breast cancer using ultrasound images: don't forget the peritumoral region. Front Oncol.

[29] Holbrook MD, Blocker SJ, Mowery YM, Badea A, Qi Y, Xu ES (2020). MRI-based deep learning segmentation and radiomics of sarcoma in mice. Tomography.

[30] Bi WL, Hosny A, Schabath MB, Giger ML, Birkbak NJ, Mehrtash A (2019). Artificial intelligence in cancer imaging: clinical challenges and applications. CA Cancer J Clin.

[31] Zhen SH, Cheng M, Tao YB, Wang YF, Juengpanich S, Jiang ZY (2020). Deep learning for accurate diagnosis of liver tumor based on magnetic resonance imaging and clinical data. Front Oncol.

[32] Tanaka T, Huang Y, Marukawa Y, Tsuboi Y, Masaoka Y, Kojima K (2020). Differentiation of small (</= 4 cm) renal masses on multiphase contrast-enhanced CT by deep learning. AJR Am J Roentgenol.

[33] Yasaka K, Akai H, Abe O, Kiryu S (2018). Deep learning with convolutional neural network for differentiation of liver masses at dynamic contrast-enhanced CT: a preliminary study. Radiology.

